# Decoupling and Decomposition Analysis of Agricultural Carbon Emissions: Evidence from Heilongjiang Province, China

**DOI:** 10.3390/ijerph19010198

**Published:** 2021-12-24

**Authors:** Qinyi Huang, Yu Zhang

**Affiliations:** 1 School of Business, Jilin University, No. 2699 Qianjin Street, Changchun 130012, China; huangqy20@mails.jlu.edu.cn; 2 School of Geographical Science, Northeast Normal University, No. 5268 Renmin Street, Changchun 130024, China

**Keywords:** grain production, agricultural carbon emissions, decoupling, LMDI

## Abstract

Ensuring food security and curbing agricultural carbon emissions are both global policy goals. The evaluation of the relationship between grain production and agricultural carbon emissions is important for carbon emission reduction policymaking. This paper took Heilongjiang province, the largest grain-producing province in China, as a case study, estimated its grain production-induced carbon emissions, and examined the nexus between grain production and agricultural carbon emissions from 2000 to 2018, using decoupling and decomposition analyses. The results of decoupling analysis showed that weak decoupling occurred for half of the study period; however, the decoupling state and coupling state occurred alternately, and there was no definite evolving path from coupling to decoupling. Using the log mean Divisia index (LMDI) method, we decomposed the changes in agricultural carbon emissions into four factors: agricultural economy, agricultural carbon emission intensity, agricultural structure, and agricultural labor force effects. The results showed that the agricultural economic effect was the most significant driving factor for increasing agricultural carbon emissions, while the agricultural carbon emission intensity effect played a key inhibiting role. Further integrating decoupling analysis with decomposition analysis, we found that a low-carbon grain production mode began to take shape in Heilongjiang province after 2008, and the existing environmental policies had strong timeliness and weak persistence, probably due to the lack of long-term incentives for farmers. Finally, we suggested that formulating environmental policy should encourage farmers to adopt environmentally friendly production modes and technologies through taxation, subsidies, and other economic means to achieve low-carbon agricultural goals in China.

## 1. Introduction

Global climate change is a key issue concerning the sustainable political, economic, social, and ecological development of governments. Greenhouse gas emissions (GHG) are an important influencing factor of global warming [1]. According to the UN Food and Agriculture Organization, agricultural carbon emissions accounted for 20% of global GHG emissions (in CO_2_ equivalents) in 2017, including emissions from livestock production and changes in land use patterns caused by the expansion of farming [2]. According to the Paris Agreement signed in 2016, a target of zero net carbon emissions and no more than 1.5 °C of warming by 2050 will be achieved. The total global use of chemical fertilizer has increased by nearly four times in the past 50 years, and large-scale carbon emissions caused by the increase in fossil energy consumption in agriculture are a key factor [3,4,5,6]. It is expected that the global population will increase by 50% by 2050, and global food demand will increase by 60–110% [7]. If traditional agricultural activity remains at current levels, the use of nitrogen and phosphorus fertilizer will increase by about 2.7–3.4 times, and nitrogen fertilizer use alone will lead to an annual equivalent emission of 3 billion tons of CO_2_ [8]. If drastic mitigation measures cannot be implemented, agricultural GHG emissions are predicted to increase by 30% by 2050, which would deteriorate the global eco-environment [9].

In view of the relationship between economy and environment, the OECD borrowed the concept of decoupling to analyze a national/regional relationship between economic growth and environmental pressure, and offered an indicator for measuring the decoupling degree, whose diagnosis is often affected by its base selection [10]. To avoid this phenomenon, Tapio constructed a decoupling index based on an elastic coefficient to reflect the sensitivity of pollutant changes to economic growth [11]. Both decoupling indexes are widely used to examine the nexus between economic growth and pollution emissions [12,13,14,15]. Currently, decoupling environmental “bads” from economic “goods” has garnered great popular support, and an increasing number of countries are adopting the idea of decoupling into policy goals [16]. 

Generally, low-carbon agriculture refers to a new mode of modern agricultural production, in which agricultural output grows steadily accompanied by low agricultural chemical inputs and low carbon emissions, by means of strengthening technology, policy, and management [17,18,19]. It has been widely accepted that environmentally friendly agricultural growth is a correct approach to achieving the goal of a green economy, and reduction in agricultural carbon emissions is conducive to improving human well-being. However, many empirical studies indicate that agricultural economic growth and agricultural carbon emissions mostly have the same increasing direction in practice [20,21,22,23,24]. For example, regarding studies dealing with agricultural carbon emissions in China, Tian et al. [25] used Tapio’s decoupling index to test the nexus between grain production and agricultural carbon emissions from 2000 to 2010, and found that strong and weak decoupling states were common from a national perspective, while other relevant conclusions from a provincial perspective varied with region [26,27]. In fact, a phase-based decoupling state at the national scale shows a mean level, and major agricultural provinces often have more contributions to agricultural carbon emissions, whose decoupling/coupling states directly affect the country’s low-carbon agricultural development. Additionally, decoupling analysis can judge the change in the economy–environment relationship, but cannot explain reasons for changes in detail. 

Decomposition analysis is frequently used for more targeted suggestions in policy terms, and the log mean Divisia index (LMDI) is widely accepted as a decomposition method, due to its advantages of complete decomposition and consistent aggregation [28,29,30,31,32]. Many scholars have used the LMDI method to decompose changes in agricultural carbon emissions, according to dividing the total changes in carbon emissions into multi-dimensional influencing factors [33,34,35,36]. In China, some scholars have decomposed agricultural carbon emissions into factors of production structure, efficiency, labor force, and agricultural economy, as well as other factors, and discovered the key factors that drive or inhibit agricultural carbon emissions [37,38,39]. Li et al. [40] decomposed the agricultural carbon emissions in Northeast China into carbon emission intensity, agricultural income, employment structure, and rural population, and found that agricultural carbon emissions in Heilongjiang province increased fastest during 1996–2013, and agricultural economic growth was the key driving factor of agricultural carbon emissions, while agricultural production efficiency was the main factor inhibiting agricultural carbon emissions. Although results of decomposition analysis change with different influencing factors, the common outcome points to agricultural economic growth, which often drives the increase in agricultural carbon emissions, while the inhibiting factors of agricultural carbon emissions vary with the indicator selection, more closely related to agricultural chemical use. Besides, it is effective for us to find the key factors based on decomposition analysis, so as to adopt targeted carbon emission reduction measures. 

China’s grain output has increased for 18 consecutive years in the 21st century, and remarkable achievements often come at the cost of agricultural resources and the environment. China’s agriculture is facing challenges of sustainable development, consumer demand change, and globalization; hard constraints include a decrease in high-quality cultivated land, and an increase in aging labor and part-time farmers, which changed the application pattern of chemical fertilizer from small amounts repeated many times to large amounts repeated fewer times [28,41,42]. Current research shows that the contribution of chemical usage on grain yield in China was more than 40%, especially chemical fertilizer, which plays an irreplaceable role in promoting agricultural production in China; it is even called the “food” of grain [43]. However, the amount of chemical fertilizer application has increased more than three times over the past 40 years, which caused about 30% of agricultural carbon emissions, and the long-term stable relationship between nitrogen and agricultural carbon emissions was more significant [44,45], especially as agricultural carbon emissions continued to grow at an average annual growth rate of 5% recently [29,30]. In this regard, green agricultural development will be the focus of China’s agricultural growth in the future [46]. Since the beginning of the 21st century, the Chinese government has issued a series of agricultural policies to encourage farmers to grow grain, and continuous bumper harvests have demonstrated the effectiveness of these policies. During this period, some environmental policies have been issued to protect the eco-environment; however, empirical studies on the effects of environmental policy in practice, especially in agricultural provinces, are insufficient. This paper takes Heilongjiang province as an example, diagnoses its status of grain production-induced carbon emissions, and provides suggestions for low-carbon agriculture on the basis of decoupling and decomposition analyses. 

## 2. Materials and Methods 

### 2.1. Study Area

Heilongjiang province is the largest agricultural province in China. The main grain crops in Heilongjiang province include corn, rice, and soybean; its agricultural mechanization level is above 98%, and its proportion of commodity grain is about 75% [31]. It is also the largest green agricultural production base in China, and its green agricultural products enjoy a national reputation. However, throughout the process of rural transformation since 2000, the hollowing out of villages is serious, and the shortage of a young and middle-aged labor force intensifies the use of chemical fertilizer and pesticide. According to statistics data from the Heilongjiang Province Statistical Yearbook (2001–2019), during the period 2000–2018, along with the years of bumper harvests in grain production (except in 2003 when farmers’ willingness to plant reached a new low), the area of cultivated land in Heilongjiang province increased from 11.8 million hectares to 15.8 million hectares, and the value added in agriculture increased from 414.4 hundred million yuan to 1463.7 hundred million yuan, more than a threefold increase. Meanwhile, the use of chemical fertilizer increased from 121.6 × 10^4^ tons to 245.6 × 10^4^ tons, and the amount of chemical fertilizer per unit area increased from 103.3 kg/hectare to 154.9 kg/hectare; the amount of pesticide used increased from 2.9 × 10^4^ tons to 7.5 × 10^4^ tons, an increase of 4.6 × 10^4^ tons over nearly 20 years. In terms of agricultural labor force, the number of agricultural labor force decreased from 744.1 × 10^4^ persons in 2000 to 609.3 × 10^4^ persons in 2018, while the aging rate of the agricultural labor force increased from 4.9% to 12.4% for the same period. All the above data show the changes in agricultural production factors in Heilongjiang province.

### 2.2. Agricultural Carbon Emissions Calculation

Following the IPCC [47] and domestic calculation methods of agricultural carbon emissions, we calculated agricultural carbon emissions and converted them into CO_2_ equivalents (CE) as Equation (1), using data from the China Rural Statistical Yearbook (2001–2019) and the Heilongjiang Statistical Yearbook (2001–2019), and adopting carbon emission coefficients from the localized research results [32,48,49,50] (Table 1). 

Equation (1) is expressed as follows:

(1) $$C=\sum_{i=1}^{i=7}T_{i}\times f_{i}$$

where *C* denotes grain production-based carbon emissions (CO_2_ equivalents, unit: tons); *i* includes 7 kinds of agricultural carbon sources: chemical fertilizer, pesticide, plastic film, electricity for irrigation, diesel for machinery, tillage, and CH_4_ emission from paddy field; *T_i_* represents the use of agricultural carbon source; *i*;$\;f_{i}$ is the carbon emission coefficient of *i* kind agricultural carbon sources.

Table 1 shows the carbon emission coefficients of each carbon source factor. 

Based on Equation (1), two indicators, that is, agricultural carbon emission intensity (*C_CI_*) and agricultural carbon density (*C_CD_*), are introduced. 

Agricultural carbon emission intensity (*C_CI_*), also called the agricultural production efficiency [38,51], is presented in Equation (2):

(2) $$C_{CI}=\frac{C}{G}$$

where *C_CI_* denotes carbon emissions per unit of value added in grain production (unit: tons/10,000 yuan), *C* is grain production-based carbon emissions (CO_2_ equivalents, unit: tons), and *G* is the value added in grain production (unit: 100 million yuan).

Agricultural carbon density (*C_CD_*) is calculated according to Equation (3):

(3) $$C_{CD}=\frac{C}{A}$$

where *C_CD_* denotes agricultural carbon emissions per unit of sown area (unit: tons/hectare), *C* is grain production-based carbon emissions (CO_2_ equivalents, unit: tons), and *A* is sown area (unit: hectare).

Data for *C_CI_* and *C_CD_* calculation were obtained from the China Rural Statistical Yearbook (2001–2019) and the Heilongjiang Statistical Yearbook (2001–2019).

### 2.3. Decoupling Index 

Following Tapio’s decoupling elasticity coefficient [11], we define the decoupling index (*DI*) as Equation (4):

(4) $$DI_{t}=\frac{\Delta C}{\Delta G}=\frac{C_{t}/C_{t-1}-1}{G_{t}/G_{t-1}-1}$$

where *DI_t_* indicates the change in one unit of CO_2_ equivalents (*C*) with respect to *G* (value added in grain production) during base period *t* − 1 and last time *t*. *C*_*t*−1_ and *C_t_* represent agricultural carbon emissions at base time *t* − 1 and last phase *t*, respectively, and Δ*C* represents the change rate of agricultural carbon emissions between last phase *t* and base time *t* − 1. *G*_*t*−1_ and *G_t_* indicate the value added in grain production in base time *t* − 1 and last phase *t*, respectively, and Δ*G* represents the growth rate of value added in grain production from last phase *t* to base time *t* − 1. Data for calculating *C_t_*, *C*_*t*−1_, *G_t_*, and *G*_*t*−1_ are from the China Rural Statistical Yearbook (2001–2019) and the Heilongjiang Statistical Yearbook (2001–2019), and carbon emission coefficients appear in Table 1. 

The relationship between grain production and agricultural carbon emissions is divided into six decoupling states on the basis of Equation (4): strong decoupling, strong coupling, weak decoupling, weak coupling, recessive decoupling, and expansive coupling (Table 2).

As shown in Table 2, strong decoupling means that the increase in agricultural carbon emissions becomes zero or negative as the value added in grain production increases; in other words, Δ*C* ≤ 0 and Δ*G* > 0, which indicates that grain production has broken away from the mode of high yield with high inputs and emissions. Strong coupling means a positive rate of change in agricultural carbon emissions (Δ*C* > 0), but a negative rate of change in value added in grain production (Δ*G* < 0). Weak decoupling means a state with positive change rates in both value added in grain production and agricultural carbon emissions, namely, Δ*C* > 0 and Δ*G* > 0, and *DI* ranges from 0 to 1. Weak coupling means that the rate of decline in agricultural carbon emissions is slower than the rate of decline in agricultural economy for the same period, namely, Δ*C* < 0 and Δ*G* < 0, and *DI* ranges from 0 to 1. Recessive decoupling means that agricultural carbon emissions decline faster than the value added in grain production declines for the same period (Δ*C* < 0 and Δ*G* < 0, and *DI* > 1), and expansive coupling means that value added in grain production rises at the cost of the increase in agricultural carbon emissions (Δ*C* > 0 and Δ*G* > 0, and *DI* > 1).

### 2.4. Log Mean Divisia Index (LMDI) Model

According to Ang [33], the LMDI method consists of multiplicative factor decomposition and additive factor decomposition, and the two can be transformed into each other. The LMDI multiplicative factor decomposition in this paper is expressed as Equation (5). The change in agricultural carbon emissions from basic period *t* − 1 to last period t can be decomposed into four factors: agricultural economy (*C_AE_*), agricultural carbon emission intensity (*C_CI_*), agricultural structure (*C_SI_*), and agricultural labor force (*C_AL_*).

(5) $$C=\frac{C}{G}\times \frac{G}{TG}\times \frac{TG}{AL}\times AL=C_{CI}\times C_{SI}\times C_{AE}\times C_{AL}\;$$

where

*C*: grain production-based carbon emissions (CO_2_ equivalents, unit: tons), calculated according to Equation (1) and carbon emission coefficients from Table 1;
*G*: value added in grain production (unit: 100 million yuan);
*TG*: total output value of agriculture (unit: 100 million yuan);
*AL*: scale of agricultural labor force (unit: 10,000 persons);
*C_AE_*: agricultural economic level, calculated by total output value of agriculture per unit of agricultural labor force (unit: yuan per capita);
*C_CI_*: agricultural carbon emission intensity, calculated by agricultural carbon emissions per unit of value added in grain production (unit: tons/10,000 yuan);
*C_SI_*: agricultural structure, value added in grain production divided by total output value of agriculture (unit: %);
*C_AL_*: scale of agricultural labor force, here, *C_AL_* = *AL* (unit: 10,000 persons).

All above data were obtained from the China Rural Statistical Yearbook (2001–2019) and the Heilongjiang Statistical Yearbook (2001–2019).

Similarly, the structural formula for the LMDI additive factor decomposition analysis is expressed in Equation (6), and Equation (6) is numerically equal to Equation (5).

(6) $$\Delta C=C_{t}-C_{t-1}=\Delta C_{CI}+\Delta C_{SI}+\Delta C_{AE}+\Delta C_{AL}\;\Delta C_{CI}=\sum^{\;}\frac{C_{t}-C_{t-1}}{LnC_{t}-LnC_{t-1}}\cdot Ln\;(\frac{C_{CI}{}_{t}}{\;C_{CI}{}_{t-1}})\;\Delta C_{SI}=\sum^{\;}\frac{C_{t}-C_{t-1}}{LnC_{t}-LnC_{t-1}}\cdot Ln\;(\frac{\Delta C_{SI}{}_{t}}{\Delta C_{SIt-1}})\;\Delta \;C_{AE}=\sum^{\;}\frac{C_{t}-C_{t-1}}{LnC_{t}-LnC_{t-1}}\cdot Ln\;(\frac{\Delta C_{AE}{}_{t}}{\;\Delta C_{AE}{}_{t-1}})\;\Delta C_{AL}=\sum^{\;}\frac{C_{t}-C_{t-1}}{LnC_{t}-LnC_{t-1}}\cdot Ln\;(\frac{\Delta C_{AL}{}_{t}}{\Delta C_{AL}{}_{t-1}})$$

Here, the change in agricultural carbon emissions from base period *t* − 1 to last period t was also decomposed into four factors: ① agricultural economic effect (Δ*C_AE_*), which reflects the change in the total output value of agriculture per unit of agricultural labor force; ② agricultural carbon emission intensity effect (Δ*C_CI_*), which indicates a comparison of the change in agricultural carbon emissions with the change in value added in grain production year by year. For example, if Δ*C_CI_* > 0, agricultural carbon emissions per unit of value added in grain production increase one year later, which offers a sign of environmental degradation; otherwise, if Δ*C_CI_* < 0, agricultural carbon emissions per unit of value added in grain production decrease one year later, which indicates environmental improvement owing to policy incentives and agricultural scientific and technological progress. Thus, Δ*C_CI_* < 0 is a sign of a low-carbon production mode; ③ agricultural structure effect (Δ*C_SI_*), which reflects the annual change in the share of the value added in grain production in the total output value of agriculture; ④ agricultural labor force effect (Δ*C_AL_*), which reflects the change in the scale of agricultural labor force within a year.

### 2.5. Data Sources and Data Processing

All data for calculating agricultural carbon emissions, decoupling analysis, and decomposition analysis were obtained from the China Rural Statistical Yearbook (2001–2019) and the Heilongjiang Statistical Yearbook (2001–2019). We took 2000 as the base period, excluded the impact of price processing according to the current year consumer price index, and obtained all the actual value added in grain and total output value of agriculture.

Table 3 reports the descriptive statistical analysis of variables. There are four indicators whose statistical data change greatly, including agricultural carbon emissions (*C*), value added in agriculture (*G*), total output value of agriculture (*TG*), and agricultural economic level (*C_AE_*). Other indicators’ statistical data are relatively stable, including agricultural carbon emission intensity (*C_CI_*), agricultural structure (*C_SI_*), and *AL* and *C_AL_* for agricultural labor force.

Before decoupling analysis, we took the value added in agriculture as the independent variable and the agricultural carbon emissions as the dependent variable, and obtained the best-fit linear equation relating these two variables (Table 4). Seen from the significance of the coefficient and R-squared, the fitting effect of the equation is good, and the relevant statistics also support the results of the regression.

## 3. Results

### 3.1. Estimation of Agricultural Carbon Emissions 

**Figure 1:**
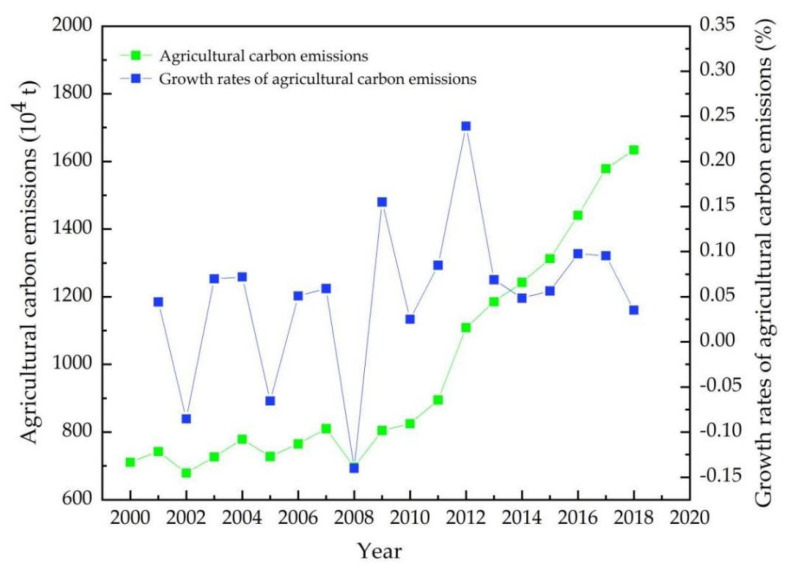
Agricultural carbon emissions in Heilongjiang province (2000–2018).

According to Equation (1), we estimated agricultural carbon emissions in Heilongjiang province during 2000–2018 (Figure 1). Agricultural carbon emissions rose from 7.1 million tons in 2000 to 16.3 million tons in 2018, including declines in agricultural carbon emissions in 2002 and 2008; agricultural carbon emissions continued to rise from 2009 to 2018. In the early stages of 2000–2008, the growth rates of agricultural carbon emissions were generally lower, and a negative growth rate occurred in 2002, 2005, and 2008; during 2009–2018, the growth rates of agricultural carbon emissions retained positive values and low volatility until recently. The policy of building a resource-conserving and environmentally friendly society was put forward in 2012, and growth rates of agricultural carbon emissions fell sharply from 25% to 4% in the following period. However, the rising trend of agricultural carbon emissions did not change during 2012–2018. 

**Figure 2:**
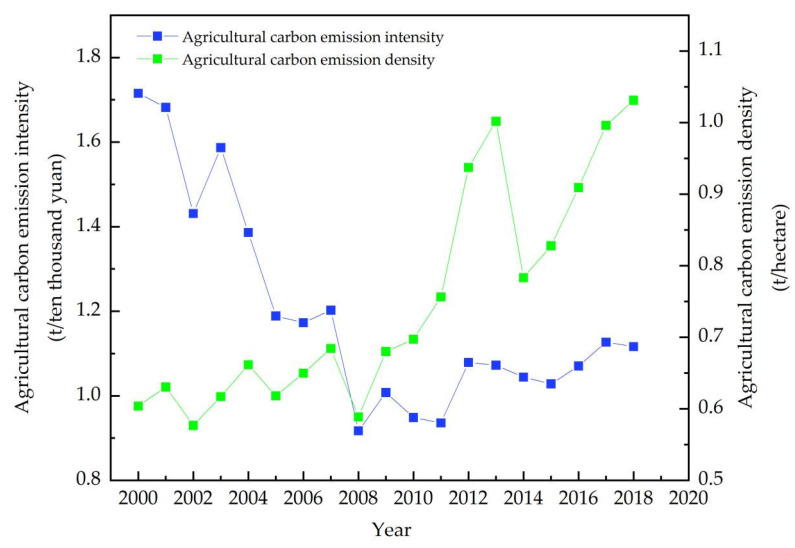
Agricultural carbon emission intensity and density in Heilongjiang province (2000–2018).

Figure 2 presents agricultural carbon emission intensity and agricultural carbon emission density in Heilongjiang province during 2000–2018. As can be seen from Figure 2, on the whole, agricultural carbon emission intensity presented a fluctuating downward trend, declining from 1.72 tons/10,000 yuan in 2000 to 1.12 tons/10,000 yuan in 2018, including a low (0.92 tons/10,000 yuan) in 2008 and subsequently fluctuating around the mean 1.0 tons/10,000 yuan over the most recent decade. Agricultural carbon emission density, however, presented a fluctuating upward trend, increasing from 0.6 tons/hectare in 2000 to 1.0 t/hectare in 2018, including early low points (such as 0.58 t/hectare in 2002 and 0.59 t/hectare in 2008). Another low point of 0.78 tons/hectare occurred in 2014 due to an increase in arable land.

The year 2008 can be regarded as a watershed. After 2008, agricultural carbon emission intensity remained at low volatility, which indicates that a low-carbon grain production mode begun to take shape in Heilongjiang province. “Action Plan for Zero Growth of Chemical Fertilizer Use by 2020” was issued in 2015, and agricultural carbon emission intensity declined accordingly. However, there was a quick rebound in the following two years, and it fell again after intensive environmental policies were issued in 2017, including several red lines defined for ecological protection, stricter national environmental protection standards, and the implementation of regulations of Environmental Protection Tax Law (draft). However, the similar environmental policy effect did not happen for agricultural carbon emission density, and agricultural carbon emission density increased during 2009–2018.

**Figure 3:**
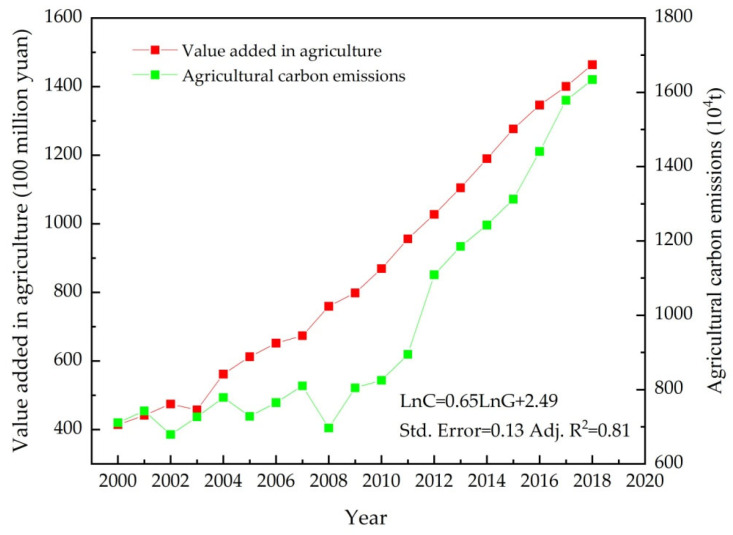
Value added in agriculture and agricultural carbon emissions in Heilongjiang province (2000–2018).

Based on the above estimated results of the regression (Table 4), the change characteristics of both grain production and agricultural carbon emissions in Heilongjiang province during 2000–2018 were further fitted as a scatter diagram (Figure 3). However, Figure 3 does not show the inner relationship between agricultural carbon emissions and agricultural economic growth. As we needed to measure the extent to which agricultural carbon emissions could decouple from grain production, decoupling analysis was essential.

### 3.2. Results of Decoupling Analysis

**Figure 4:**
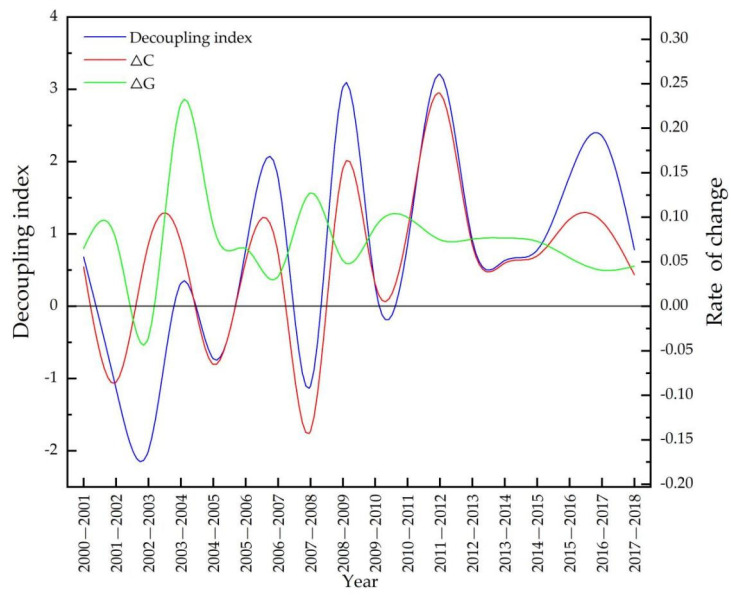
Decoupling index in Heilongjiang province during 2000–2018.

On the basis of Equation (4) and Table 2, the annual results of decoupling analysis are shown in Table 5 and Figure 4. Four decoupling states occurred in Heilongjiang province during 2000–2018: strong decoupling (for 3 years: 2002, 2005, and 2008), weak decoupling (for 9 years), expansive coupling (for 5 years), and strong coupling (for 1 year). 

According to Table 5, the sum of strong decoupling and weak decoupling states amounted to 12 years during the study period, and weak decoupling occurred the most (half of the study period), which overall was a better indication of the relationship between grain production and agricultural carbon emissions. Table 5 shows that the change rates in agricultural growth almost always had a rising tendency, except in 2003 when change in the value added in grain production fell to −3.5%, due to farmers being less willing to grow a small amount of grain, while the change rate in agricultural carbon emissions was a positive value (Δ*C* was 7% for the same period). As a result, this was the only strong coupling state that occurred, and a series of policies supporting agriculture and farmers have been issued since 2004. Under the influence of an environmental policy issued in 2012, weak decoupling state, strong decoupling state, and expansive coupling state alternately appeared for the rest of the study period. 

Figure 4 indicates that the decoupling index (*DI*) frequently fluctuated, and four kinds of decoupling or coupling states appeared irregularly. Even after the environmental policy issued in 2012, this situation did not improve, for example, weak decoupling states happened continuously in 2013, 2014, and 2015, followed by expansive coupling states in 2016 and 2017. A weak decoupling state occurred in 2018, but we could not guarantee that a weak or strong decoupling would happen next. Heilongjiang province still faces opportunities and pressure for low-carbon agricultural development. 

Table 5 and Figure 4 show the temporal change characteristics of the relationship between grain production and agricultural carbon emissions in Heilongjiang province during 2000–2018. The details of this that actually drive the decoupling state have not been established; therefore, further decomposition analysis of influencing factors is essential for more instructive suggestions and to achieve the goal of decoupling in Heilongjiang province. 

### 3.3. Results of LMDI Decomposition 

**Figure 5:**
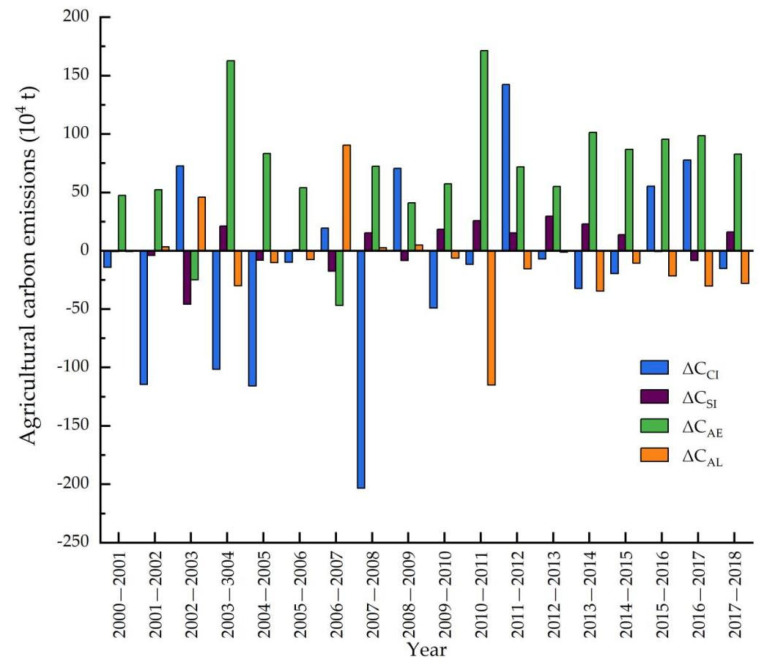
Contributions of each factor to agricultural carbon emissions in Heilongjiang province (2000–2018).

On the basis of Equations (5) and (6), the decomposition results of agricultural carbon emissions in Heilongjiang province during 2000–2018 are shown in Table 6 and Figure 5. Using the LMDI method, annual increases in the total agricultural carbon emissions (Δ*C*) were a common phenomenon during the study period, except for three important time nodes: 2002, 2005, and 2008, consistent with the time when a strong decoupling state occurred in Section 3.2.

As shown in Table 6 and Figure 5, agricultural economic effect (Δ*C_AE_*) and agricultural structure effect (Δ*C_SI_*) were two factors that drove the increase in agricultural carbon emissions overall during 2000–2018, and the agricultural economic effect (Δ*C_AE_*) was more important and exceeded the agricultural structure effect (Δ*C_SI_*) by far, with contributions of 12.61 and 0.85 million tons of agricultural carbon emissions, respectively. In detail, Δ*C_AE_* almost always presented a positive driving factor of an increase in agricultural carbon emissions except in 2006–2007, which indicated that agricultural growth had strong momentum in agricultural carbon emissions in Heilongjiang province; it also revealed the difficulty of inhibiting agricultural carbon emissions as the largest agricultural province in China.

In terms of factors inhibiting agricultural carbon emissions, the agricultural carbon emission intensity effect (Δ*C_CI_*) and agricultural labor force effect (Δ*C_AL_*) contributed –2.57 and –1.64 million tons of agricultural carbon emissions in reducing agricultural carbon emissions, respectively. According to the change trend of the agricultural carbon emission intensity effect, Δ*C_CI_* frequently varied, and its driving or inhibiting factor of agricultural carbon emissions was not stable. Additionally, agricultural labor force (Δ*C_AL_*) showed a clear inhibiting effect from 2010. The combined effects of agricultural carbon emission intensity (Δ*C_CI_*) and agricultural labor force (Δ*C_AL_*) were generally not enough to balance the agricultural economic effect (Δ*C_AE_*). Four decomposition factors varied in the quantity and driving direction of agricultural carbon emissions during 2000–2018.

Below, we discuss the results of integrating decoupling analysis with decomposition analysis.

First, strong decoupling states occurred in three years: 2002, 2005, and 2008. On the one hand, the agricultural growth rate of the strong decoupling state was a positive value (Δ*G* > 0), while the change rate of agricultural carbon emissions was a negative value (Δ*C* < 0). On the other hand, the agricultural economic effect (Δ*C_AE_*) was a positive value, which drove the increase in agricultural carbon emissions, while the agricultural carbon emission intensity effect (Δ*C_CI_*) was a negative value, which inhibited agricultural carbon emissions. Upon further analysis, we found that the inhibiting power of Δ*C_CI_* exceeded the driving power of Δ*C_AE_* in agricultural carbon emissions in the same period, which proved the key inhibiting role of the agricultural carbon emission intensity effect (Δ*C_CI_*) in neutralizing agricultural carbon emissions driven by the agricultural economic effect (Δ*C_AE_*). From a policy perspective, environmental policies issued in 2002, 2005, and 2008 indeed had an effect on inhibiting agricultural carbon emissions. For example, the Clean Production Promotion Law was drafted in 2002; the Kyoto Protocol signed by the Contracting Parties to the United Nations Framework Convention on Climate Change officially came into force in 2005, China’s National Plan to Address Climate Change was introduced, and Xi Jinping first put forward the idea that lucid waters and lush mountains are invaluable assets; the Clean Production Promotion Law and the Circular Economy Promotion Law were promulgated in 2008. Accordingly, agricultural carbon emission intensity played a prominent part in inhibiting agricultural carbon emissions in these years and led to the occurrence of strong decoupling.

Second, weak decoupling states happened mostly in Heilongjiang province during the study period. Similar to the strong decoupling state, the agricultural growth rate was a positive value (Δ*G* > 0); and changes in agricultural carbon emissions were also positive values (Δ*C* > 0). Results of decomposition analysis showed that the agricultural economic effect in each weak decoupling state was a positive value (Δ*C_AE_* > 0), which drove the increase in agricultural carbon emissions, while agricultural carbon emission intensity effect was a negative value (Δ*C_CI_* < 0), which inhibited agricultural carbon emissions from increasing, similar to the strong decoupling state to some extent. However, unlike the strong decoupling state, the driving power of Δ*C_AE_* was stronger than the inhibiting power of *C_CI_* in agricultural carbon emissions in the same period, the inhibiting power of agricultural carbon emission intensity effect is insufficient to contend with agricultural economic effect, which results in weak decoupling. Additionally, both the agricultural structure effect (Δ*C_SI_*) and agricultural labor force (Δ*C_AL_*) did not appear to have a stable variation tendency. 

Third, regarding expansive coupling states, the agricultural carbon emission intensity effect was a stable driving factor of the increase in agricultural carbon emissions (Δ*C_CI_* > 0), while the agricultural economic effect appeared to be more of a driving factor of the increase in agricultural carbon emissions (Δ*C_AE_* > 0). The agricultural structure effect was largely an inhibiting factor of agricultural carbon emissions (Δ*C_SI_* < 0), and the agricultural labor force effect (Δ*C_AL_*) did not display a clear driving or inhibiting effect on agricultural carbon emissions.

Lastly, a strong coupling state occurred only once in 2003, due to lacking long-term incentive policies of grain growing, which resulted in a negative agricultural economic effect (Δ*C_AE_*) and agricultural structure effect (Δ*C_SI_*), with contributions of −25.03 × 10^−4^ and −45.79 × 10^−4^ tons of agricultural carbon emissions, respectively. The agricultural carbon emission intensity effect (Δ*C_CI_*) and agricultural labor force effect (Δ*C_AL_*), however, acted as driving factors of the increase in agricultural carbon emissions, with contributions of 72.51 × 10^−4^ and 45.79 × 10^−4^ tons of agricultural carbon emissions, respectively. As a result, agricultural growth fell and agricultural carbon emission intensity rose, causing the worst result of strong coupling.

## 4. Discussion and Policy Suggestions

The goal of low-carbon agricultural development focuses both on the stability and continuity of agricultural economic growth, and on the carrying capacity of agricultural resources and the environment. Therefore, the effective intervention of agricultural environmental policy, and the full participation of agricultural scientific and technical measures are needed for low-carbon agriculture [26].

During the last 20 years, Heilongjiang province has achieved a better agricultural economic growth effect, which fits the status of the largest agricultural province. From the perspective of policy effect, agricultural policies have been successful in encouraging farmers to grow grain since 2004; however, there is still much room for improvement in environmental policy. As the largest green agricultural production base in China, the low-carbon grain production mode has begun to take shape in Heilongjiang province according to the change in agricultural carbon emissions intensity after 2008 and the results of decoupling analysis. In fact, except in 2002, 2005, and 2008, when strong decoupling states occurred, the Chinese government regularly issued environmental policies in the 21st century. However, both coupling states following a decoupling state and decoupling state alternating with coupling state indicated that environmental policy had strong timeliness and weak persistence, probably mainly due to the lack of long-term incentives for farmers.

Seen from international experience, the agricultural policies of Europe, the United States, Japan, and other countries keep agricultural environmental protection in focus. At the same time, the formulation of environmental policy should pay close attention to economic development, and encourage agricultural producers to take the initiative to adopt environmentally friendly production modes and technologies through taxation, subsidies, and other economic means. In practice, the current environmental policy in China pays more attention to the environmental effect and ignores the economic effect on agricultural development, and problems of low compensation standard and insufficient compensation are common. The adoption of environmentally friendly technology itself poses a certain risk, so insufficient incentive levels will lead to a lack of enthusiasm for the subject.

Considering the key inhibiting effect of agricultural carbon emission intensity based on the above decomposition analysis, this paper provides the following suggestions from the perspectives of agricultural scientific and technical measures, agricultural environmental policy, and incentive measures for farmers.

(a). We will upgrade agricultural science and technology to promote agricultural carbon emission reduction in grain production. Measures include: ① adopting soil testing formula fertilization, and improving the efficiency of agricultural chemical usage and utilization, so as to reduce the problem of excessive application of chemical fertilizer from carbon sources; ② promoting diversified agricultural modernization means, such as water and fertilizer integration, slow-release and long-acting fertilizers, nitrification inhibitors, and other emission reduction technologies and new fertilizers; ③ strengthening the research and development of low-toxicity and low-pollution agricultural chemical materials, such as the development of high-efficiency compound fertilizers, low-toxicity pesticides, and low-cost degradable agricultural film.
(b). We will integrate agricultural subsidy policy with environmental policy. Conversational tillage mode and returning straw to the field have significant effects on enhancing soil fertility, improving grain production efficiency, and increasing grain yield, which also help to reduce the use of chemical fertilizer. We have seen the Comprehensive Implementation Plan of Straw Utilization in Heilongjiang Province issued in November 2021, which published detailed implementation rules for paying incentives to farmers who return straw to the field. It will benefit all farmers to adopt these new agricultural activities, under the incentive mechanism design.
(c). We will encourage new business entities, such as large growers, nongovernmental service organizations, and leading enterprises, to widely apply green prevention and control technologies. Agricultural technical training and precision skill training for farmers should be strengthened. Additionally, establishing a strict quality and safety supervision traceability system in society and a pricing mechanism, adopting an appropriate incentive mechanism to compensate farmers, can directly or indirectly inhibit the use of chemical fertilizer, pesticide, and other chemicals, all which help to reduce agricultural carbon emissions.

## 5. Conclusions 

In this paper, we adopted decoupling and decomposition analysis to examine the relationship between grain production and agricultural carbon emissions in Heilongjiang province during 2000–2018. 

The results of decoupling analysis indicated that four decoupling states occurred: weak decoupling, strong decoupling, expansive coupling, and strong coupling. Although decoupling states, including strong and weak decoupling, appeared frequently, there was no sign of a stable evolution path from coupling to decoupling, which highlights both the pressure and challenges for Heilongjiang province as it develops towards low-carbon agriculture. 

Using the LMDI method, we decomposed the change in agricultural carbon emissions into four factors: agricultural economic effect (*C_AE_*), agricultural carbon emission intensity (*C_CI_*), agricultural structure effect (*C_SI_*), and agricultural labor force effect (*C_AL_*). The results show that the agricultural economic effect (Δ*C_AE_*) and agricultural structure effect (Δ*C_SI_*) were two factors generally driving the increase in agricultural carbon emissions during 2000–2018; the agricultural economic effect (Δ*C_AE_*) was the most important driving factor. The agricultural carbon emission intensity effect (Δ*C_CI_*) and agricultural labor force effect (Δ*C_AL_*) were frequent factors inhibiting agricultural carbon emissions, and the agricultural carbon emission intensity effect (Δ*C_CI_*) was the key inhibiting factor.

Further integrating decoupling with decomposition analyses, we found that a low-carbon grain production mode began to take shape in Heilongjiang province after 2008, and the existing environmental policies had strong timeliness and weak persistence, probably due to the lack of long-term incentives for farmers. Based on this, specific suggestions for low-carbon agricultural development were provided.

## Figures and Tables

**Table 1:** Carbon emission coefficients.

Carbon Sources	Emission Factor	Reference
Fertilizer	1.53 kg CE/kg (N fertilizer); 1.63 kg CE/kg (P fertilizer); 0.66 kg CE/kg (K fertilizer)	[32]
Pesticide	0.20 kg CE/kg (Herbicide); 16.60 kg CE/kg (Insecticide)	[32]
Plastic film	22.7 kg CE/kg	[32]
Electricity for irrigation	1.23 kg CE/kWh^−1^	[48]
Diesel for machinery Tillage	0.89 kg CE/kg 312.6 kg CE/km^2^	[32] [49]
CH_4_ emissions from paddy field	66.2 kg CH_4_/hm^2^	[50]

**Table 2:** Degrees of decoupling states.

Decoupling States	Relationship between Agricultural Carbon Emissions and Grain Production
Weak coupling	Δ*C* < 0, Δ*G* < 0, 0 < *DI* < 1
Strong coupling	Δ*C* > 0, Δ*G* < 0, *DI* < 0
Expansive coupling	Δ*C* > 0, Δ*G* > 0, *DI* > 1
Recessive decoupling	Δ*C* < 0, Δ*G* < 0, *DI* > 1
Weak decoupling	Δ*C* > 0, Δ*G* > 0, 0 < *DI* < 1
Strong decoupling	Δ*C* ≤ 0, Δ*G* > 0, *DI* ≤ 0

**Table 3:** Descriptive statistical analysis of variables.

Variable	Unit	N	Mean	Min	Max	Standard Deviation
*C*	10,000 tons	19	982.28	678.99	1633.97	319.86
*G*	100 million yuan	19	867.40	414.40	1463.70	349.42
*TG*	100 million yuan	19	1303.92	625.10	2076.74	472.88
*AL*	10,000 persons	19	702	609	781	57
*C_AE_*	yuan per capita	19	19,114.86	8400.75	34,086.75	8282.29
*C_CI_*	tons/10,000 yuan	19	1.20	0.92	1.72	0.25
*C_SI_*	%	19	0.66	0.61	0.70	0.03
*C_AL_*	10,000 persons	19	702	609	781	57

**Table 4:** Estimated results of the regression model.

Variable	Coefficient	Std. Error	t-Statistic	Prob
Constant	2.49	0.49	5.11	0.00
lnG	0.65	0.07	8.94	0.00
**Statistic**				**Value**
R-squared				0.82
Adjusted R-squared				0.81
S.E. of regression				0.13
Sum squared resid				0.29
Log likelihood				12.90
F-statistic				79.94
Prob (F-statistic)				0.00
Mean dependent var				6.84
S.D. dependent var				0.30
Akaike into criterion				−1.15
Schwarz criterion				−1.05
Hannan–Quinn criterion				−1.13
Durbin–Watson stat				0.52

**Table 5:** Decoupling states in Heilongjiang province (2000–2018).

Year	Δ*C* (%)	Δ*G* (%)	*DI*	Decoupling States
2000–2001	0.044	0.065	0.682	Weak decoupling
2001–2002	−0.085	0.075	−1.138	Strong decoupling
2002–2003	0.070	−0.035	−1.998	Strong coupling
2003–2004	0.072	0.227	0.317	Weak decoupling
2004–2005	−0.065	0.090	−0.727	Strong decoupling
2005–2006	0.051	0.065	0.784	Weak decoupling
2006–2007	0.059	0.033	1.775	Expansive coupling
2007–2008	−0.140	0.127	−1.102	Strong decoupling
2008–2009	0.155	0.051	3.033	Expansive coupling
2009–2010	0.025	0.089	0.282	Weak decoupling
2010–2011	0.085	0.100	0.850	Weak decoupling
2011–2012	0.239	0.075	3.206	Expansive coupling
2012–2013	0.069	0.075	0.912	Weak decoupling
2013–2014	0.048	0.077	0.631	Weak decoupling
2014–2015	0.056	0.073	0.775	Weak decoupling
2015–2016	0.098	0.054	1.795	Expansive coupling
2016–2017	0.095	0.041	2.355	Expansive coupling
2017–2018	0.035	0.045	0.781	Weak decoupling

**Table 6:** Decomposition of agricultural carbon emissions in Heilongjiang province during 2000–2018 (10^4^ t).

Year	Δ*Cc_I_*	Δ*Cs_I_*	Δ*C_AE_*	Δ*C_AL_*	Δ*C*
2000–2001	−14.26	−0.73	47.31	−0.83	30.76
2001–2002	−114.70	−4.07	52.07	3.36	−63.46
2002–2003	72.51	−45.79	−25.03	45.79	47.49
2003–2004	−101.56	21.14	162.74	−29.97	52.35
2004–2005	−115.84	−8.25	83.23	−10.09	−50.95
2005–2006	−9.93	0.70	53.94	−7.64	37.07
2006–2007	19.43	−17.66	−46.80	90.26	45.23
2007–2008	−203.57	15.17	72.28	2.56	−113.56
2008–2009	70.60	−8.49	41.02	4.81	107.94
2009–2010	−49.23	18.42	57.33	−6.28	20.24
2010–2011	−11.76	25.83	171.20	−115.28	69.99
2011–2012	142.17	15.40	71.80	−15.41	213.96
2012–2013	−7.07	29.60	54.96	−1.14	76.35
2013–2014	−32.29	22.97	101.35	−34.69	57.34
2014–2015	−19.69	13.65	86.84	−10.63	70.17
2015–2016	55.32	−0.83	95.35	−21.59	128.25
2016–2017	77.57	−8.28	98.44	−30.25	137.48
2017–2018	−15.24	16.14	82.66	−27.93	55.63
2000–2018	−257.55	84.93	1260.72	−164.96	923.13

## Data Availability

China Rural Statistical Yearbook (https://data.cnki.net/trade/yearbook/single/n2019120190?z=z009), and Heilongjiang Statistical Yearbook (http://tjj.hlj.gov.cn/tjsj/tjnj/202001/t20200120_76425.html).
